# Impact of perioperative nutritional status on the outcome of abdominal surgery in a sub-Saharan Africa setting

**DOI:** 10.1186/s13104-017-2765-8

**Published:** 2017-09-18

**Authors:** Christian Gael Mambou Tebou, Mazou N. Temgoua, Agnès Esiene, Blondel Oumarou Nana, Jean Jacques Noubiap, Eugène Sobngwi

**Affiliations:** 1 Higher Institute of Health Sciences, Université des Montagnes, P.O Box 208, Bagangté, Cameroon; 2 Department of Internal Medicine and Specialties, Faculty of Medicine and Biomedical Sciences, P.O Box 1364, Yaoundé, Cameroon; 3 Anesthesiology and Intensive Care Department, Central Hospital of Yaoundé, P.O Box 87, Yaoundé, Cameroon; 4 National Social Insurance Fund Health Center of Yaoundé, P.O Box 5777, Yaoundé, Cameroon; 5 Department of Medicine, Groote Schuur Hospital and University of Cape Town, Cape Town, 7925 South Africa; 6 National Obesity Center, Central Hospital of Yaoundé, P.O Box 87, Yaoundé, Cameroon

**Keywords:** Abdominal surgery, Nutritional status, Perioperative period

## Abstract

**Background:**

Malnutrition is a clinical condition of multifactorial etiologies and it is associated with several adverse outcomes. In high-income countries, malnutrition has been described as a determinant of delayed wound healing, surgical site infections and mortality in the postoperative period. There is limited information available regarding the outcome of surgery in malnourished patients in sub-Saharan Africa.

**Methods:**

A cross-sectional analytic study was carried out between March and August 2014 in the visceral surgery and the emergency departments of the Yaounde Central Hospital in Cameroon. All consecutive consenting preoperative and postoperative patients of abdominal surgical procedures were enrolled. Variables studied were: socio-demographic characteristics, medical and surgical past histories, nutritional survey, anthropometric parameters and serum albumin level in order to determine the nutritional risk index (or Buzby score).

**Results:**

A total of 85 patients aged from 19 to 50 years with mean age of 34.4 ± 8 years were included. The most performed abdominal surgical procedure was appendectomy (30.6%). The prevalence of preoperative malnutrition according to the Buzby score was 39.1%. Mean postoperative weight lost was 2.9 ± 1.2 kg and mean decrease in postoperative serum albumin was 4.2 ± 0.2 g. A normal postoperative serum albumin was associated with a favorable outcome [OR (95% CI) = 55 (13.4–224.3), p < 0.001].

**Conclusions:**

The prevalence of malnutrition is high in our visceral surgery and emergency departments; this is associated with an increased risk of adverse early postoperative outcomes. Overall, our results emphasize the need of optimizing perioperative care through routine nutritional assessment and management of surgical patients in Cameroon.

**Electronic supplementary material:**

The online version of this article (doi:10.1186/s13104-017-2765-8) contains supplementary material, which is available to authorized users.

## Background

Malnutrition is due to an imbalance between food intakes and needs, resulting in several disorders like immunosuppression, increased susceptibility to infections, delay wound healing, increased drug intolerance and death [1]. In low and middle-income countries, malnutrition has mainly been described as a significant determinant of the under-five mortality [2]. On the other hand, in high-income countries the emphasis of its complications have been laid more on the adult population with a reported malnutrition prevalence of 20–40% in hospital setting [3]. In surgery, malnutrition has been found to be an independent predictor of morbidity and mortality in postoperative patients [4]. Current recommendations stipulate screening of malnutrition and risk stratification for all patients in the preoperative period, in a bid to prevent related complications [5]. Much more, nutritional support following surgery has been well codified in several universally validated programs including the “clinical pathway”, “fast track” or “accelerate recovery programs” [4]. The postoperative mortality of abdominal surgery in the sub-Saharan African setting is still high [6]. However, data on the effect of malnutrition on postoperative outcomes of abdominal surgery are scarce in sub-Saharan African populations. This study aimed to assess the perioperative nutritional status of patients undergoing abdominal surgery and to determine the relationship between perioperative nutritional status and postoperative outcomes in a tertiary hospital in Cameroon.

## Methods

### Study population

This was a cross sectional analytic study conducted between March and August 2014, in the Visceral Surgery and the Emergency Surgery Units of the Central Hospital of Yaounde, Cameroon. We systematically included all consecutive consenting patients aged between 19 and 59 years, either in the preoperative and postoperative period of abdominal surgery.

### Data collection

For each participant, using a structured questionnaire, we collected data on sociodemographic characteristics, medical and surgical past history, nutritional survey, weight change in the last 6 months and gastrointestinal symptoms lasting for at least 2 months. All the participants underwent a complete physical examination. We measured weight in light clothes with a Seca Scale balance to the nearest 0.1 kg, and height using a calibrated stadiometer. Weight was measured on the admission time and everyday of hospitalization until discharge. The weight used to appreciate postoperative nutritional status was that measured on the day of discharge.

Blood samples were collected for the measurement of pre-and postoperative serum albumin levels by the bromocresol green colorimetric method. Serum albumin was measured at the time of admission and discharge. All participants were followed during hospitalization until discharge in the visceral surgery department.

### Definitions

Patients were classified according to the body mass index (BMI) (kg/m^2^) WHO classification 2007 [7] as follows: underweight (<18.5), normal range (18.5–24.9), overweight (25–29.9) and obesity (≥30). The nutritional risk index also called the Buzby index was calculated as follows:

$$1.519\,[\text{Serum}\,\text{albumin}] + 0.417\frac{Actual\,weight}{Ideal\,weight}$$

By this index, all patients were classified into three categories of malnutrition namely; mild (>97.5), moderate (83.5–97.5) and severe (<83.5) [8].

Minor abdominal procedure was defined as any abdominal surgery which is generally not associated with long postoperative fasting period.

A favorable evolution was a condition when we had: good wound healing observed after 2 days, normal resumption of oral feeding, normal ambulation, and stable hemodynamic parameters persistent for more than 48 h prior to discharge, and absence of death.

### Statistical analysis

Data analysis was done using the SPSS version 20 software. Chi square and Fisher exact tests were used to compare qualitative variables. Quantitative variables were studied by the Student test. The association between nutritional status and postoperative outcomes was expressed by odds ratio with their 95 percent confidence intervals. Linear regression was used to establish the relationship between weight loss and length of hospitalization. The correlation coefficient r was used to express this relationship. A *p* value <0.05 was considered statistically significant (Additional file 1).

## Results

### Participants

A total of 121 participants were approached, we excluded 36 patients because of insufficient data, and finally 85 subjects were retained. Because of emergencies conditions, complete data on nutritional status was available only for 23 patients in preoperative setting. Female represented 55.3% (n = 47) (sex ratio = 0.80). The mean age was 34.9 ± 8.2 (range 19–50) years. The mean length of hospitalization was 9.88 ± 3.64 days. Appendicitis was the most common abdominal surgical indication in 30.6% (n = 26) of cases (Table 1).

**Table 1:** General characteristic of the study population

Characteristics	Number (n = 85)	Percentage
Gender		
Male	38	44.7
Female	47	55.3
Indications of surgery		
Appendicitis	26	30.6
Evisceration	2	2.4
Anal wound	3	3.5
Perineum gangrene	2	2.4
Hemoperitoneum post blunt abdominal with splenic rupture	2	2.4
Digestive hemorrhage	3	3.5
Hernia	9	10.5
Parietal mass	2	2.4
Intestinal occlusion	12	14.1
Peritonitis	15	17.6
Post ulcer bulbar stenosis	3	3.5

### Preoperative nutritional status

Before surgery 69.6% (n = 16/23) of participants had a normal BMI, 21.7% (n = 5/23) were overweight and 8.7% obese (n = 2/23). Low serum albumin levels (<35 g/l) were observed in 17.4% (n = 4/23) of participants. A low preoperative serum albumin level was significantly associated with patient’s age ≥ 40 years (p < 0.001).

Using the nutritional risk index, 30.4% (n = 7/23) and 8.7% (n = 2/23) of participants had moderate and severe malnutrition respectively.

### Postoperative nutritional status

After surgery, 16.5% (n = 14/85) of participants had malnutrition according to the BMI and almost half of participants (44.7%, n = 38/85) had low serum albumin levels. Likewise, the proportion of moderate and severe malnutrition increased in postoperative period (43.5%, n = 37/85 and 23.5%, n = 20/85 respectively). The male gender was significantly associated to postoperative malnutrition according to the BMI (p < 0.01).

### Postoperative outcome

**Fig. 1:**
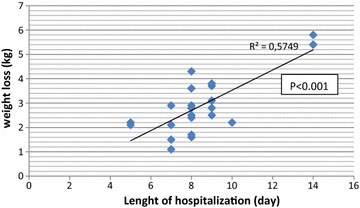
Correlation between weight loss and length of hospitalization

During hospitalization, the mean weight loss was 2.9 ± 1.2 kg and there was a relationship between the lengths of hospital stay and weight loss (p < 0.001 and r^2^ = 0.5749) (Fig. 1). A total of 61.2% of participants had a favorable postoperative outcome.

The nutritional parameters associated with a favorable evolution were: normal postoperative BMI [OR (95% CI) = 2.9 (1.19–7.34), p = 0.018], normal postoperative level of serum albumin [OR (95% CI) = 55 (13.49–224.32), p < 0.001] and normal nutritional risk index [OR (95% CI) = 19.3 (2.18–169.79), p = 0.003] (Table 2).

**Table 2:** Relation between nutritional status and postoperative evolution

	Value	Favorable/unfavorable	OR (95% CI)	p value
Postoperative BMI	18.5–24.9	31/11	2.95 (1.19–7.34)	0.018
Postoperative albuminemia	35–50	44/3	55 (13.49–224.32)	<0.001
Preoperative Buzby Score	>97.5	11/2	19.25 (2.18–169.79)	0.003

*OR* odd ratio, *CI* confidence interval, *BMI* body mass index

## Discussion

The main objective of this study was to evaluate the nutritional status and postoperative outcome in patients undergoing abdominal surgery. There was a high frequency of malnutrition in the study population. One-third of participants had unfavorable postoperative outcomes. The nutritional parameters associated with a favorable outcome were: normal postoperative BMI, and normal postoperative serum albumin.

This study was an observational study, in order to be aligned with the ethical principle of non-maleficence. Participants were selected in a single hospital with the aim to standardize its surgical and postsurgical intensive care practices. The variability of nutritional index in this study enhanced the chance to detect any nutritional risk. The prevalence of preoperative malnutrition according to the Buzby score was 39.1%, this is inferior to the 53.3% reported by Rakotondrainibe et al. in 2013, probably because their study sample included a higher proportion of major abdominal surgical procedures known to have long postoperative fasting time [9]. In our series, the predominance of minor abdominal surgical procedures was already highly suggestive that there should be high prevalence of malnutrition. Shpata et al., in 2014 in a study done in Tirana found a higher prevalence of preoperative malnutrition of 65.3% in patients undergoing gastrointestinal surgery [10]. It is normal to have more case of malnourished patients when the type of surgery is purely gastrointestinal, because the postoperative fasting time is sometime prolonged [11]. In gastrointestinal cancer surgery this prevalence is higher with a value reach 84.9%, this is explained by the hypercatabolic state of patients [10]. Patients with gastrointestinal malignancies were excluded from our sample size in order to reduce selection bias. Low level of serum albumin has been found in 17.4% of participants contrary to the 27.3% found by Rakotondrainibe et al. in group patients who underwent gastrointestinal cancer surgery [9]. This difference is because of the predominance of major abdominal procedures which have high risk of malnutrition.

Similarly to Wu et al. in 2005, we found that the prevalence of malnutrition increased in the postoperative period explained by the physiological hypercatabolic state which occurs during this period. In our series, the male gender was a determinant of postoperative malnutrition. This observation is in line with that reported by Aparecida et al. in 2014 [12].

The good nutritional status observed in this study was correlated with a favorable postoperative outcome at 1 week; probably because, nutrients help to reduce infection, accelerate healing and general rehabilitation. The benefit of an optimal perioperative nutritional status have been well cited in studies carried out in high-income countries [13]. Current guidelines recommend that all nutritional grade 2 (NG2) patients should have nutritional advice and supplementation prior to surgery. The NG4 patients on the other hand, should have enteral or parenteral nutritional assistance for at least 7 days before surgery [14]. However these guidelines are not well codified in sub-Saharan Africa.

We acknowledge some limitations of our study. Its single study setting and the small sample of surgical procedures (n = 83), as such our results may not be generalized to all abdominal surgical procedures. Also, the data concerning surgical pathology were taken in the medical file of the patients and sometimes these were not clear enough. The reported surgical pathologies required minor abdominal procedures, contrary to the studies done in western populations. However, to the best of our knowledge, it’s the first study in our milieu, and this may serve as base for future study.

## Conclusion

Our findings suggest that patients undergoing abdominal surgery have poor nutritional status, further exacerbated in the postoperative period. This would need to be further explored in large multicentre studies in our setting. Due to the risk of potential postsurgical complications from malnutrition and the management challenges akin to resource-limited settings, we highlight the need to integrate a routine nutritional assessment program in the management of patients undergoing abdominal surgery, in a bid to improve the postsurgical outcome.

## Additional file

**Additional file 1.** Supplemental material.

## Abbreviations

BMI: body mass index
CI: confidence interval
HIV: human immunodeficiency virus
kg: kilograms
NG: nutritional grade
OR: odd ratio
SPSS: Statistical Package for Social Sciences
WHO: World Health Organization
