# Supplementary material for: Impact of perioperative nutritional status on the outcome of abdominal surgery in a sub-Saharan Africa setting
Source: BMC Res Notes. 2017 Sep 18;10:484. doi: 10.1186/s13104-017-2765-8 (PMC5604173; doi:10.1186/s13104-017-2765-8)
Supplement: Supplementary file 1 — Additional file 1. Supplemental material. [file 13104_2017_2765_MOESM1_ESM.docx]

**Questionnaire**

Inclusion date…/…./…

**Sociodemographic characteristics**

Age (years)……………………….

Sex: 1= M 2= F

Profession…………………………

**Past History**

Cancer: 1= Yes 2= No

Chronic Kidney Disease: 1= Yes 2= No

HIV: 1= Yes 2= No

Diabetes: 1= Yes 2= No

Use of Corticosteroids: 1= Yes 2= No

Limbs amputation: 1= Yes 2= No

Abdominal surgery: 1= Yes 2= No

Others: 1= Yes 2= No

If yes, precise……………………………………..

**Nutritional survey**

Are you on particular diet? 1= Yes 2= No

If yes, precise………………………..

Are you perceived a weight loss during the last 6 months? 1= Yes 2= No

Are you developed one of these symptoms during the last 2 months?

Nausea 1= Yes 2= No

Vomiting 1= Yes 2= No

Diarrhea 1= Yes 2= No

Anorexia 1= Yes 2= No

**Surgical indications**……………………………………………...

**Physical and biological examination**

D0 (admission): weight (kg) = Height (cm)= Albumin level (g/l)=

**Weight on hospitalization**

D1: D7: D13: D19:

D2: D8: D14: D20

D3: D9: D15: D21:

D4: D10: D16: DX:

D5: D11: D17:

D6: D12: D18:

**Discharge’s time…/…. /…….**

**Parameters on discharge’s time:**  weight (kg) = Albumin level (g/l)=

**Evolution on hospitalization:** 1= Favorable 2= Unfavorable
